# Chalcones—Features, Identification Techniques, Attributes, and Application in Agriculture

**DOI:** 10.3390/molecules29102247

**Published:** 2024-05-10

**Authors:** Magdalena Dziągwa-Becker, Marta Oleszek, Sylwia Zielińska, Wiesław Oleszek

**Affiliations:** 1 Department of Weed Science and Tillage Systems, Institute of Soil Science and Plant Cultivation, State Research Institute, Orzechowa 61, 50-540 Wrocław, Poland; 2 Department of Biochemistry and Crop Quality, Institute of Soil Science and Plant Cultivation, State Research Institute, Czartoryskich 8, 24-100 Puławy, Poland; moleszek@iung.pulawy.pl (M.O.); wieslaw.oleszek@iung.pulawy.pl (W.O.); 3 Division of Pharmaceutical Biotechnology, Department of Pharmaceutical Biology and Biotechnology, Wroclaw Medical University, Borowska 211, 50-556 Wrocław, Poland; sylwia.zielinska@umw.edu.pl

**Keywords:** chalcones, biopesticides, herbicides, insecticides, low-risk active substances

## Abstract

This review article is a comprehensive and current overview on chalcones, covering their sources, identification methods, and properties with a particular focus on their applications in the agricultural sector. The widespread use of synthetic pesticides has not only led to increased resistance among weeds and pests, resulting in economic losses, but it has also raised significant health concerns due to the overuse of these chemicals. In line with the European Green Deal 2030 and its Farm to Fork strategy, there is a targeted 50% reduction in the use of chemical pesticides by 2030, emphasizing a shift towards natural alternatives that are more environmentally sustainable and help in the restoration of natural resources. Chalcones and their derivatives, with their herbicidal, fungicidal, bactericidal, and antiviral properties, appear to be ideal candidates. These naturally occurring compounds have been recognized for their beneficial health effects for many years and have applications across multiple areas. This review not only complements the previous literature on the agricultural use of chalcones but also provides updates and introduces methods of detection such as chromatography and MALDI technique.

## 1. Introduction

**Figure 1:**
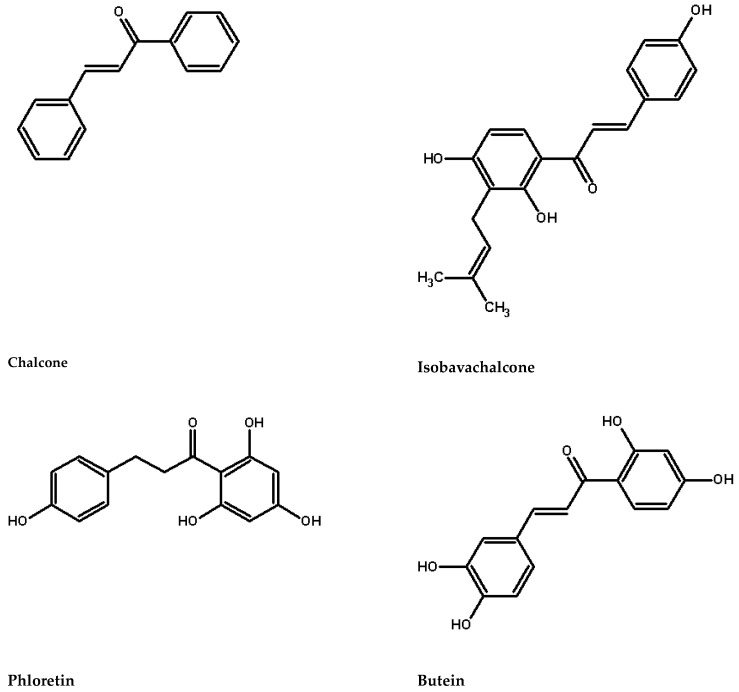
Chalcones structures.

Chalcones are distinguished by their lack of a C ring in the basic flavonoid skeleton structure (Figure 1), classifying them as open-chain flavonoids. These compounds are well-known specialized (secondary) metabolites found ubiquitously throughout the plant kingdom, playing crucial roles in plant growth and defense against pathogens. Flavonoids, a class of phenolic compounds, are categorized based on the oxidation level of the heterocyclic ring and the presence of hydroxyl or methyl groups on the benzene ring into 12 subgroups: anthocyanins, aurones, chalcones, dihydroflavonols, flavanones, flavones, flavanols, isoflavones, leucoanthocyanidins, phlobaphenes, proanthocyanidins, and stilbenes [1].

The term chalcone derives from the Greek word “chalcos”, meaning bronze. Prominent chalcone examples include phloridzin, butein, phloretin, and chalconaringenin. These compounds are prevalent in strawberries, berries, certain wheat products, tomatoes, pears, apples, citrus fruits, and hops. Chalcones and their derivatives have attracted considerable attention due to their numerous nutritional and biological activities [2,3,4,5], including anti-inflammatory, antitumoral, antibacterial, antifungal, antimalarial, antitubercular, and antipigmentation properties, often yielding excellent results [4,6,7,8]. Moreover, chalcones can be useful in weed control [9,10]. Interestingly, a single compound such as isobavachalcone may exhibit multiple activities, including chemopreventive, anti-cancer, anti-bacterial, and anti-fungal properties [11,12]. Historically utilized in traditional medicine, naturally occurring chalcones are now finding new applications [13]. With the European Union’s Farm to Fork strategy targeting a reduction in synthetic pesticide use by 2030, biopesticides are increasingly popular not only for their differing modes of action but also for their enhanced environmental sustainability. This review provides an updated overview on characteristic, properties, methods of detection, and applications in agriculture of chalcones.

## 2. Characteristic

Chemically, chalcones are *α*,*β*-unsaturated ketones consisting of two aromatic rings (known as ring A and ring B) connected by a three-carbon alkenone unit [14]. In higher plants, these compounds are biosynthesized through the action of the enzyme chalcone synthase (CHS, EC2.3.1.74), which converts one molecule of *p*-coumaroyl-CoA and three molecules of malonyl-CoA. CHS is not only crucial not only for plant development but is also activated under stress conditions such as UV exposure, wounding, and herbivory and microbial attacks, leading to the synthesis of secondary metabolites like phenolic compounds [15,16]. Chalcones, with their simple structural scaffold, are found in many naturally occurring compounds, but a large number of novel chalcone structures have also been synthesized over the years [17]. Advances in synthetic chemistry have produced bioactive synthetic chalcone derivatives that have been structurally optimized to reduce toxicity, allowing their use in medicine, the chemical industry [14], food production, and agriculture [18]. Since the early 19th century, numerous researchers have developed synthetic chalcones, e.g., Kostanecki and Tambor, who first synthesized a chalcone using o-acetoxychalcone dibromides and alcoholic alkali [14,19]. Synthetic methods include Claisen–Schmidt condensation, using hydrochloric acid, synthesis from phosphonate carbanion, microwave- and solvent-free synthesis involving biocatalysts, and aldol condensation synthesis using (hetero)aryl methyl ketones and 4(benzyloxy)benzaldehyde [12,20,21].

During extraction, native chalcone glycosides tend to transform to flavanone glycosides, limiting their occurrence in food [3]. Mixtures of retrochalcones along with isomeric flavanones and chalcones such as liquiritigenin and isoliquiritigenin have been identified in licorice root (*Glycyrrhiza* spp.) and some licorice-based traditional medicines [22]. Dihydrochalcones (DHC), which are characteristic of apples and their products, are most commonly represented by phloridzin [23]. Peeled fruits contain fewer DHCs, as they are primarily located in the peel. Conversely, commercially produced juices and ciders have a 5–10-times higher content of DHC not only because the whole fruit is used but also due to the thermal treatment that inactivates the enzymes responsible for DHC degradation [24].

## 3. Chalcones Identification

### 3.1. Sample Preparation

One of the most effective sample preparation methods prior to chromatographic analysis, such as UPLC-MS/MS, is freeze drying. Specifically, freeze-dried samples can be processed using a mixer mill with a zirconia bead for 1.5 min at 30 Hz. Subsequently, 100 mg of lyophilized powder is dissolved in 1.2 mL of a 70% methanol solution, vortexed for 30 s every 30 min for a total of six cycles, and then refrigerated at 4 °C overnight. Following centrifugation at 12,000 rpm for 10 min, the extracts are filtered prior to UPLC-MS/MS analysis [25]. For further purification of the crude product, column chromatography can be employed, eluting with a petroleum ether/EtOAc mixture (3:1, *v*/*v*) to isolate the desired compounds [4]. Krauze-Baranowska et al. [26] optimized the SPE-HPLC method for analyzing chalcones in various species and clones of *Salix* utilized in the pharmaceutical sector. The process involved the drying and grinding 1 g of bark, which was then extracted with methanol (3 × 30 mL) for 45 min in 60°. The combined methanolic extracts were concentrated under reduced pressure. Then, 80 μL of the concentrated extract was evaporated to dryness and re-dissolved in an equal volume of 20% can for solid-phase extraction (SPE). Similarly, Guvenalp et al. [27] employed methanol as an extractant for isolating bioactive compounds, including two novel chalcone glycosides, from mint. The aerial parts of the plant, air-dried and powdered (1000 g), were extracted four times with methanol at 40 °C. Following vacuum evaporation, the crude extract was dissolved in water and subjected to sequential liquid–liquid partitions with petroleum ether, CHCl_3_, EtOAc, and n-butanol. The solvents were then evaporated under reduced pressure using a rotary evaporator [28]. Chen et al. [28] extracted powdered *Fructus psoreleae* using methanol solution acidified with hydrochloric acid as an extraction solvent, aided by ultrasonication. Afterwards, the mixture was centrifuged at 3000 g for 20 min, and the supernatant was collected for further analysis.

### 3.2. Liquid Chromatography Coupled with Absorbance Detectors

Chalcones exhibit two primary absorption bands: Band I typically ranges from 340–390 nm, while band II is found between 220–270 nm.

Pobłocka-Olech [29] reported on the qualitative and quantitative HPLC analysis of a flavonoids mixture, which included naringenin, naringenin (+) and (-)-5-*O*-glycosides, naringenin 7-*O*-glycoside, isosalipurposide, and its *p*-coumaric ester. This analysis was performed on a Discovery C18 column using gradient elution with a mobile phase of acetonitrile and water adjusted with orthophosphoric acid. Detection was achieved using a UV–vis detector at 280 nm and a diode array detector (DAD), enhancing identification speed with the incorporation of the solid-phase extraction (SPE) method [30]. Isosalipurposide, also known as phloridzin chalcone, is identified as a monosaccharide derivative of trans-chalcone substituted with hydroxy groups at positions 4, 4′, and 6 and a *β*-D-glucopyranosyloxy group at position 2′. It functions as a plant metabolite and an antioxidant [31].

Krauze-Baranowska et al. [26] detailed the separation of chalcones from willow tree bark using a Discovery C18 column (5 μm, 150 × 2.1 mm). They employed a 15 min gradient elution at a flow rate of 0.4 mL/min, identifying chalcones and flavanones via UV–vis DAD detection at 280 nm. Quantification was achieved using external standardization employing isoliquiritigenin and its derivative, 6″-*O*-*p*-coumaroyl ester, as reference substances [27]. Additionally, Chen et al. [28] conducted HPLC–UV analyses on a DL-C_l8_ column (5.0 μm, 250 mm, and 4.6 mm) with a flow rate of 0.5 mL/min using acetonitrile (A) and 0.01 M formic acid (B) as a mobile phase. Their method utilized gradient elution with detection at 246 nm, providing detailed insights into the chromatographic behavior of these compounds.

### 3.3. Two-Dimensional High-Performance Liquid Chromatography (2D-HPLC)

Pobłocka-Olech [29] demonstrated that rapid comparative analysis of willow bark can be efficiently conducted using a two-dimensional high-performance liquid chromatography (2D-HPLC) system. This method incorporated 52 standard substances for the chromatographic separation, comprising 29 phenolic acids, 21 flavonoids (including 9 flavonols, 4 flavones, 4 flavanones, 2 biflavones, and 2 chalcones), as well as salicin and catechin. The separation process utilized an on-line system via a heart-cut technique. The first dimension (I) involved a Supelcosil LC-18 column with gradient elution, increasing the concentration of methanol in a methanol/water mixture at a flow rate at 0.4 mL/min. The second dimension (II) used monolithic silica gel-packed Chromolith Performance RP18e column, with isocratic elution employing acetonitrile and water mixtures as eluents. Under these conditions, methanol extracts from barks of *Salix purpurea*, *S. daphnoides* clone, and *S. sachalinensis* ‘Sekka’ were analyzed. The 2D-HPLC technique allows for the analysis of plant extracts without the need for prior purification, making it a valuable tool for identifying secondary metabolites in various plant matrices.

### 3.4. Infrared Spectroscopy, FTIR, and HNMR

The infrared (IR) spectra of chalcones reveal distinct features characteristic of their molecular structure. The asymmetric and symmetric stretching vibrations of the aromatic C–H bonds are evident in the ranges of 3120–3080 cm^−1^ and 3060–3040 cm^−1^, respectively, each marked by two low-intensity bands. Additionally, the C–H stretching band of the =C–H group is observed at 3030–3010 cm^−1^. Vibrations of the aromatic rings are assigned to bands at 1610–1570 cm^−1^, while the in-plane deformation of the =C–H bond appears as a broad weak band at 1460–1430 cm^−1^. The carbonyl stretching vibrations in the enones (=C–C=O) are located between 1650 and 1685 cm^−1^ [30].

Further, a range of chalcone derivatives can also be analyzed using spectroscopic techniques such as Fourier transform infrared spectroscopy (FTIR) and proton nuclear magnetic resonance (1HNMR) [4,31]. According to Hassan et al., the FTIR spectra of these derivatives show distinct peaks due to the carbonyl chalcone C=O stretching at 1708 and 1712 cm^−1^ and the C=C stretching of alkenes at 1612 and 1622 cm^−1^. The 1HNMR analysis of derivative C identifies protons of the amine group at 10.7 ppm and 10.6 ppm, protons of the aromatic ring between 7.5–6.6 ppm, and protons associated with HC-S at 5 ppm. In derivative D, the protons of the amine group appear at 10.5 ppm, aromatic ring protons are found in the range of 7.7–6.8 ppm, f HC-S protons were at 4.8 ppm, and methyl group protons were at 2.2 ppm [31].

### 3.5. Liquid Chromatography Coupled with Mass Spectrometry

Zou et al. [25] analyzed extracts of *Paeonia delavayi* var. *lutea* using a Shimadzu UPLC-ESI-MS/MS system, operating under the following conditions: the UPLC column, Agilent SB-C18 (1.8 µm, 2.1 × 100 mm); the mobile phase consisted of pure water with 0.1% formic acid and acetonitrile with 0.1% formic acid. The samples were analyzed with a gradient program and an injection volume of 4 μL, and the effluent was fed into an ESI-triple quadrupole-linear ion trap (QTRAP)-MS [25], whereas, Ma et al. [32] developed a selective high-performance liquid chromatography–mass spectrometry (LC-MS/MS) method for quantifying isobavachalcone (IBC) in rat plasma. Neobavaisoflavone was used as an internal standard (IS). The analytes were separated on a Kinetex C18 column (2.6 μm, 100 mm × 2.1 mm i.d., Phenomenex) using isocratic elution with mobile phase of acetonitrile:water (60:40, *v*/*v*) at a flow rate of 0.2 mL/min. An electrospray ionization (ESI) source was applied and operated in the negative ion mode, and quantification was achieved through multiple reactions monitoring (MRM), with target fragment ions at *m*/*z* 323.0 → 118.9 for IBC and *m*/*z* 321.1 → 265.0 for the IS. This method demonstrated good linearity within the concentration range of 3.79–484.5 ng/mL for IBC in rat plasma [32]. Furthermore, Chen et al. [28] detected major constituents including bakuchiol, bavachin, bavachinin, and isobavachalcone in *Fructus psoraleae* using HPLC coupled with UV, MS, and electrochemical detectors (ECD). The MS analysis was performed in negative ion mode utilizing selected ion monitoring (SIM), offering high selectivity and sensitivity for determining the constituents within a mass range of 50–1000 *m*/*z*.

### 3.6. MALDI Technique

According to Krittanai et al. [33], liquid chromatography coupled with UV detection exhibits low sensitivity for identifying licochalcone A (LicoA), a compound found in the root of Chinese licorice (*Glycyrrhiza inflata* Batalin). Consequently, an enzyme-linked immunosorbent assay (ELISA) was developed, using a specific antibody, to quantitatively measure LicoA. This assay was validated to be highly specific to LicoA with minimal cross-reactivity to structurally similar substances. Following method optimization, the detection limit was established at 4.32 ng/mL, and the quantification range was set between 6.84–107.21 ng/mL. The newly developed assay was then successfully applied to measure LicoA concentration in both raw licorice and commercially available products.

## 4. Properties

### 4.1. Chalcones Biological Activities and Their Applications in Agriculture

Chalcones are highly bioactive compounds that play a crucial role in agriculture for weeds and pests control. These eco-friendly pesticides and weed control agents demonstrate extensive biological effects and are effective against a variety of organisms [6].

The biological activity of chalcones largely depends on their structure, particularly the number and position of substituents like hydroxyl groups and the α,β double bond [13]. Moreover, the modification of chalcones by attaching specific functional groups can enhance their desired activities. Due to their unique structure, chalcones also serve as intermediate in the synthesis of therapeutically valuable compounds [34]. Effective in controlling weeds and pests, chalcones exhibit a range of activities, including phytotoxic, bactericidal, antifungal, antiviral, antihelmintic, insecticidal, and antifeedant properties.

### 4.2. Herbicides and Plant Growth Regulators

Chalcones are increasingly recognized in agricultural for their phytotoxic activities, which are instrumental in the development of new herbicides. Research indicates that many chalcones display strong herbicidal effects while maintaining low toxicity to crops [6,9]. Their activity varies based on the substituents on rings A and B of their structure, the concentrations used, and the specific plant species and organs targeted. Derivatives containing functional groups such as phenoxyacetic acid, 4-(N,N-dimethylamino)phenyl, N-methylpyrrole, and particularly thiophenyl have demonstrated significant inhibitory effects [10,13].

Chotsaeng et al. [10] found that flavokawains, chalcone-related derivatives of xantoxyline, significantly inhibit the growth of Chinese amaranth and barnyard grass. Among 45 synthesized chalcones, (E)-2-(2-(3-Oxo-3-(thiophen-2-yl)prop-1-enyl)phenoxy)acetic acid emerged as the most potent, credited to the thiophenyl and phenoxyacetic acid groups on rings A and B, respectively.

Further studies, such as those by Perera et al. [35], revealed that other chalcone derivatives, namely salsolol A and B, are effective against *Lemna pausicotata*, with IC50 values of 261, 275, and 251 µM, respectively.

Yun et al. [36] described one phytotoxicological mechanism where chalcones suppress plant growth by inhibiting the activity of coenzyme A ligase (4CL), which is crucial for lignin monomer biosynthesis. Similarly, Nguyen et al. [37] identified chalcones as selective inhibitors of phosphoenolpyruvate carboxylase (PEPC), a key enzyme for carbon fixation and biomass increase in the C4 photosynthetic pathway of many damaging weeds.

Diaz-Tielas et al. [38] explored another mechanism where transchalcone induces programmed cell death (PCD) in *Arabidopsis* seedlings by altering mitochondrial function, leading to membrane depolarization and the release of PCD-inducing factors. This supports chalcone’s potential as a plant-growth regulator.

Moreover, Diaz-Tielaz et al. [9] investigated the selectivity of chalcone’s phytotoxic effects between crops and weeds. They examined both spraying and watering applications on matured *Arabidopsis* and found that trans-chalcone adversely affects the germination and early root growth of some weeds and crops, but it is beneficial for others.

In additional research, phloretin, a well-known dihydrochalcone, exhibited significant dose-dependent growth retardation, severe morphological abnormalities, and agravitropic behavior in *Arabidopsis* seedlings [39]. These findings further underscore the potential of chalcones as effective and selective agents in plant growth regulation and weed control.

### 4.3. Fungicides

Chalcones are renowned for their antifungal properties against a wide range of human fungal pathogens. Their mechanism of action involves inhibition ß(1,3)-glucan and chitin synthases—enzymes responsible for synthesizing ß(1,3)-glucan and chitin polymers in fungal cell walls, respectively [40].

Additionally, numerous studies have demonstrated the effectiveness of chalcones against plant pathogens that cause significant economic losses on arable land globally.

For instance, research by Svetaz et al. [41] showed that *Phomopsis longicolla* exhibits considerable sensitivity to chalcones derived from *Zuccagnia punctata*. The chloroform fraction of an ethanolic extract from this plant, which includes compounds like 2′,4′-dihydroxy-3′-methoxychalcone and 2′,4′-dihydroxychalcone, displayed strong activity against *P. longicolla* Hobbs CE117 (MIC = 6.25 and 3.12 mg mL^−1^, respectively) and *Colletotrichum truncatum* CE175 (MIC = 6.25 mg). These fungi are major contributors to soybean diseases, adversely affecting seed quality and yields due to their high incidence and persistence. Further, Badaracco et al. [42] found that a plant-origin chalcone, 1,3-difenylo-2propen-1on, inhibited fungi such as *Alternaria* sp., *P. longicolla*, *Fusarium proliferatum*, and *Fusarium subglutinans,* which are pathogens of significant agronomic and food importance. The minimum inhibitory concentrations (MIC) ranged from 62.5 to 125 μg mL^−1^, with fungicidal activity noted against *Alternaria* sp. and *P. longicolla* at minimum fungicidal concentrations (CFM) of 125 and 250 μg mL^−1^, respectively. Additionally, a study by Oleszek et al. [43] highlighted the potent antifungal activity of methanolic extract from apple pomace, particularly a fraction rich in phloridzin, a well-known chalcone, against pathogens like *Botrytis* sp., *Fusarium oxysporum*, *Petriella setifera*, and *Neosartorya fischeri.*

The natural occurrence of chalcones has inspired the synthesis of novel synthetic chalcones with enhanced antifungal properties. Recent advancements include research by Chen et al. [4], who tested chalcone derivatives containing pyridazine against nine fungi: *Rhizoctonia solani*, *Botrytis cinerea*, *Phomopsis* sp., *Colletotrichum acutatum*, *Botryosphaeria dothicdea* (Bd), *Fusarium graminearum* (FG), *Colletotrichum gloeosporioides*, *Sclerotinia sclerotiorum* (SS), and *Phytophthora capsica*. These derivatives displayed stronger antifungal activity than azoxystrobin, the standard control agent, suggesting their potential as future fungicides through disruption of fungal cell membranes and inhibition of growth.

Further exploration into synthetic chalcone derivatives by Zhou et al. [44] involved compounds containing piperazine tested against *Rhizoctonia solani* and *Colletotrichum gloeosporioides*. The observed mechanism embraced an induction of irregular and shriveled growth of mycelium and a rupture of the mycelium surface, highlighting the diverse potential of chalcone structure in combating fungal infections.

### 4.4. Antiviral Agents

Chalcones and their derivatives has also been explored as antiviral agents, displaying significant efficacy against viruses such as the tomato ringspot virus (ToRSV). Initial studies, like those on *Chenopodium quinoa*, identified 2-hydroxychalcone as a modest inhibitor of ToRSV [45]. Moreover, Onyilagha et al. [46] examined the antiviral capabilities of 21 different chalcones, revealing that antiviral properties were enhanced by hydroxylation at the 2′, 3′, and 4′ positions of the A-ring and the C-4′ position of the B-ring but were reduced by hydroxylation at C-5′ and methoxylation of the B-ring.

Recent agricultural literature primarily addresses the impact of chalcone derivatives on the tobacco mosaic virus (TMV), noting that the type, position, and nature of substituents within the chalcone structure are crucial for their antiviral effectiveness.

Dong et al. [47] synthesized and tested a series of novel chalcone derivatives incorporating a 1,1-dichloropropene moiety and found that many of them exhibited moderate-to-strong antiviral activity, with one derivative showing inactivation activity against TMV comparable to ningnanmycin, a commercially available antiviral agent.

Zhou et al. [48] reported that the incorporation of purine and benzenesulfonamide moieties into chalcone molecules resulted in effective antiviral activity against both TMV and cucumber mosaic virus (CMV), significant crop pathogens. One particular compound was notably effective, attributed to an electron-donating group at the 2 position of benzenesulfonamide aromatic ring and minimal steric hindrance. This compound demonstrated strong binding affinity to the coat protein (CP) of TMV, a critical functional protein involved in various viral processes such as translation, transcription, and self-assembly of TMV. The number of hydrogen bonds formed played an important role in the stabilizing the interaction with TMV-CP, enhancing antiviral activity.

In another recent study, Zhang et al. [49] explored the anti-TMV activity of chalcone derivatives containing a pyrimidine base. These compounds showed superior curative activity, with EC50 values significantly lower than those of ningnanmycin. The effectiveness of these derivatives varied depending on the nature, position, and length of the substituent chains, underscoring the complex interplay between chalcone structure and antiviral efficacy.

### 4.5. Nematicides

Plant-parasitic nematodes represent a significant threat to agriculture, with increasing resistance to existing nematicides and pressing need for innovative solutions. Research on the nematocidal properties of chalcones, particularly concerning the *Meloidogyne* genus, which includes some of the most economically damaging phytopathogens, has been gaining momentum. In pursuit of new nematocidal agents, researchers have developed a series of fused-ring compounds derived from chalcones using a ring closure design strategy. These compounds have shown promising activity against *M. incognita*.

Silva et al. [50] evaluated twelve synthesized chalcone analogues and identified (1*E*,4*E*)-1,5-di(4-nitrophenyl)-2-butylpenta-1,4-dien-3-one as having superior activity compared to the commercial nematicide Carbofuran^®^. This compound demonstrated a remarkable reduction of 51% in galls and 68% in eggs when applied to tomato plants infected with nematodes. Its mechanism of action is believed to involve the inhibition of P450 enzyme, which plays a critical role in the oxidation of several substances within the nematode.

Studies by Cao et al. [51] showed that chalcones-like compounds containing a 2-carbonyl tiophene group exhibited excellent nematocidal activity. The most potent of these compounds achieved an impressive LC50 value of 3.20 mg/L within 72 h in vitro and inhibited nematode activity by 100.00% at a concentration of 40 mg/L in the test matrix.

Additionally, Attar et al. [52] reported polarity as well as planarity in the effectiveness of chalcone compounds against the nematode *Caenorhabditis elegans*. They found that organic chalcones, being less polar than their synthesized ferrocenyl (Fc) analogues, were also more active. This increased activity is linked to chalcone’s ability to penetrate the cell walls of the organisms more effectively. These findings underscore the potential of structurally diverse chalcone derivatives as potent nematocidal agents, offering new avenues for controlling nematode populations that threaten crop health and productivity.

### 4.6. Insecticides

Numerous studies have demonstrated the effectiveness of both natural and synthesized chalcones as insecticides. Among the naturally occurring chalcones, xanthohumol and isoxanthohumol, isolated from hop (*Humulus lupulus* L.), are good examples. These compounds have shown significant insecticidal activity against the peach-potato aphid (*Myzus persicae*) [53].

Shakil and Saxena [54] isolated a new chalcone, i.e., cordifolin, from woody stem of giloe (*Tinospora cordifolia*), and evaluated its effect on larvae of *Spodoptera litura*. The findings indicated that cordifolin caused delays in pupation, prolonged the pupal period, and reduced pupal weight.

Extensive research has also been conducted on synthesizing and developing new chalcones and their derivatives with insecticidal properties, focusing on structural modifications and the selection of appropriate substituents. Hidalgo et al. [55] studied both bis- and mono-chalcones against *Spodoptera frugiperda*, finding that two mono-chalcones with bromines and hydroxyl groups on ring A and a N-N dimethyl group on ring B killed 40% and 60% of larvae, respectively, when incorporated to the larval diet at a concentration of 100 mg per g. In contrast, bis-chalcones did not exhibit such activity. However, Devi et al. [56] reported contradictory results, where bis-chalcones demonstrated greater toxicity than mono-chalcones. The study by Kumar et al. [8] is particularly noteworthy, as it is the first report on the pesticidal activity of chalcones against *Plutella xylostella*, synthesized using microwave irradiation. It was found that an electron-withdrawing group on ring A of the chalcone was crucial for pesticidal activity, while ring B could accommodate either electron-withdrawing or electron-donating substituents. In particular, chlorine (Cl) substitutions and their specific positions on both rings were critical. The compound 1,3-Bis(4-chlorophenyl)prop-2-en-1-one exhibited the highest activity, with the LC50 value of 170.24 µg/mL. These results lay a foundations for further modifications and the design of novel chalcone-based pesticidal agents targeting *P. xylostella* and similar insect pests.

## 5. Conclusions

In summary, chalcones, both natural and synthetic, represent a versatile and complex group of compounds with a broad spectrum of biological activities. Known for centuries, the exploration of their potential is ongoing, with many of their pharmacological benefits still underexplored and poorly understood. This makes them a subject of significant interest within the scientific community. This review highlights the herbicidal, fungicidal, antiviral, insecticidal, and plant-growth regulating properties of various chalcones, offering a comprehensive overview and introducing detection methods for the first time, all based on the latest literature.

Given that plants do not produce chalcones in large quantities, extracting them from natural sources poses a challenge. Additionally, their relatively short half-life further complicates their study and use. These factors have spurred intensive research into the synthesis and further development of chalcones. Furthermore, there is a critical need for in-depth studies to elucidate the mechanisms of action of reactive chalcones, evaluate their efficacy in real-world agricultural settings, and assess their environmental and human safety. These investigations are essential for harnessing the full potential of chalcones and ensuring their responsible utilization.

## Data Availability

Not applicable.
